# The role of the microbiome in NAFLD and NASH

**DOI:** 10.15252/emmm.201809302

**Published:** 2018-12-27

**Authors:** Aleksandra A Kolodziejczyk, Danping Zheng, Oren Shibolet, Eran Elinav

**Affiliations:** ^1^ Immunology Department Weizmann Institute of Science Rehovot Israel; ^2^ Department of Gastroenterology The First Affiliated Hospital Sun Yat‐sen University Guangzhou China; ^3^ Department of Gastroenterology and Liver Disease Tel Aviv Sourasky Medical Center Tel Aviv Israel; ^4^ Sackler Faculty of Medicine Tel Aviv Sourasky Medical Center Tel Aviv Israel

**Keywords:** microbiome/microbiota, nonalcoholic fatty liver disease, nonalcoholic steatohepatitis, Digestive System, Microbiology, Virology & Host Pathogen Interaction

## Abstract

Nonalcoholic fatty liver disease (NAFLD) is the hepatic manifestation of cardiometabolic syndrome, which often also includes obesity, diabetes, and dyslipidemia. It is rapidly becoming the most prevalent liver disease worldwide. A sizable minority of NAFLD patients develop nonalcoholic steatohepatitis (NASH), which is characterized by inflammatory changes that can lead to progressive liver damage, cirrhosis, and hepatocellular carcinoma. Recent studies have shown that in addition to genetic predisposition and diet, the gut microbiota affects hepatic carbohydrate and lipid metabolism as well as influences the balance between pro‐inflammatory and anti‐inflammatory effectors in the liver, thereby impacting NAFLD and its progression to NASH. In this review, we will explore the impact of gut microbiota and microbiota‐derived compounds on the development and progression of NAFLD and NASH, and the unexplored factors related to potential microbiome contributions to this common liver disease.

**Glossary**
Bacteremia: Presence of bacteria in the blood.
Bile acids: A group of molecules synthesized from cholesterol in hepatocytes that are major component of the bile. They function in facilitation of nutrient absorption in the gut and metabolic signaling in several tissues in the body.
Dysbiosis: Imbalance in composition of microbiota that has a negative effect on the physiology of the host.
Fecal microbiota transplantation (FMT): Medical procedure that involves transfer of fecal matter from a donor or donors into the intestinal tract of recipient.
Gut epithelial barrier: A layer of epithelial cells of the gut that forms a physical barrier between contents of the gut lumen and the host while allowing transport of desired molecules such as nutrients.
Gut–liver axis: The interaction between physiology of the gut and the liver that is based on the physical connection between these organs via portal vein.
Histone deacetylases (HDACs): Family of enzymes that cleave acetyl group from histones resulting in more positive charge of histone tails and tighter binding of histones to negatively charged DNA.
Inflammasome: A term describing multiprotein complexes formed in response to variety of signals such as pathogens as well as sterile stress signals and result in secretion of pro‐inflammatory signals such as IL‐1β or IL‐18 and eventually induction of pyroptosis.
Intestinal bacterial outgrowth: Abnormally high amount of bacteria present in the intestine.
Kupffer cells: Macrophages of the liver.
NAFLD: Presence of at least 5% hepatic steatosis in patients without excessive alcohol consumption, including the entire spectrum of fatty liver disease ranging from simple fatty liver to nonalcoholic steatohepatitis, fibrosis, and cirrhosis.
NASH: Presence of at least 5% hepatic steatosis with histological characterization of liver inflammation and hepatocyte injury with or without fibrosis.
Oxidative stress: A situation when there are more free radicals produced than the cell can neutralize with antioxidants. In this situation, free radicals interact and damage cellular components such as proteins, lipids, or nucleic acids.
Reactive oxygen species (ROS): Unstable, very reactive molecules containing oxygen, such as peroxide, superoxide, hydroxyl radical.
S‐adenosyl methionine (SAM) cycle: Reactions that produce, consume, and regenerate S‐adenosyl methionine. Regeneration of SAM allows it to function as a key donor of methyl group in the cell.
Short‐chain fatty acids: Fatty acids with less than six carbon atoms.
Steatosis: A process defined by accumulation of excessive amount of lipids in the liver cells, mainly inside the hepatocytes.

## Introduction

### NAFLD and NASH

Nonalcoholic fatty liver disease (NAFLD) is defined as the presence of at least 5% hepatic steatosis but lacking common causes of secondary hepatic fat accumulation, such as excessive alcohol consumption, chronic viral hepatitis, autoimmune hepatitis, congenital hepatic disorders, or long‐term use of steatosis‐inducing medications (Chalasani *et al*, 2018). NAFLD often, but not always, occurs together with type 2 diabetes, obesity, dyslipidemia, and hypertension, which constitute cardiometabolic disease (Younossi *et al*, 2016).

The global prevalence of NAFLD is estimated to be 25.24%, with the highest rates in the Middle East (31.79%) and South America (30.45%), followed by Asia (27.37%), North America (24.13%), Europe (23.71%), and Africa (13.48%; Younossi *et al*, 2016). The incidence of NAFLD is reported to range from 20 to 50 cases per 1,000 person‐years in different countries (Chalasani *et al*, 2018). These alarming numbers make NAFLD a major clinical and economic burden and the new epidemic in global chronic liver disease (Younossi *et al*, 2018). A substantial portion of NAFLD patients develop nonalcoholic steatohepatitis (NASH), which is histologically characterized by the presence of hepatic inflammation and liver injury. NAFLD, and particularly NASH, can potentially progress to fibrosis, cirrhosis, and eventually hepatocellular carcinoma (Pais *et al*, 2013; Calzadilla Bertot & Adams, 2016). In this context, NAFLD is currently the leading cause of chronic liver disease in the USA and Europe. Indeed, NASH‐associated cirrhosis has become the second leading indication for adult liver transplantation in the USA and continues to grow (Wong *et al*, 2015). Current treatment of NAFLD focuses on lifestyle modification. Liver‐targeted pharmacotherapy is reserved for high‐risk patients and is mostly experimental (Tilg, 2017; Chalasani *et al*, 2018).

The pathogenesis of NAFLD is poorly understood. It is thought to involve complex interactions among genetic susceptibility variants, environmental factors, insulin resistance, and changes in the gut microbiota (Arab *et al*, 2017). The interplay between these factors results in altered lipid metabolism and excessive lipid accumulation in hepatocytes, culminating in the development of NAFLD. Furthermore, the microbiota is involved in alternating the balance between pro‐inflammatory or anti‐inflammatory signals, thereby contributing to inflammation that may result in progression to NASH. There is thus an urgent need to fully understand the pathogenesis of NAFLD, and the contribution of the microbiome to NAFLD and its progression to NASH, which may enable to improve diagnostics, patient stratification, and identification of new therapeutic targets.

### The gut microbiome

Recent years brought enhancing understanding that the microbiota, an ecosystem consisting of bacteria, archaea, protists, fungi, and viruses living in the human gut, is not an idle bystander but an interconnected, active player in human physiology. The microbiota composition and function are shaped by a variety of host and environmental factors including diet, physical activity, medication, circadian activity, and geographical localization. This complex community of microorganisms bears several folds more genetic information than the human genome, for example, enzymes capable of biochemical functions lacking in the human host, such as deconjugation of primary bile acids or breakdown of indigestible carbohydrates. This person‐specific microbiota configuration cooperates with the unique host genetics in contributing to individualized traits and phenotypes.

The gut microbiota actively participates in the digestion of food and facilitates the absorption of the dietary molecules into the portal and systemic circulation (Jumpertz *et al*, 2011; Ridaura *et al*, 2013), interacts with the mucosal immune system, to shape development of its antigen recognition, recruitment, proliferation, and effector function, and may impact the host even at far sites from where it actually resides, via modulation of migrating immune cells and through metabolites influxing from the gut into the systemic circulation, where they can affect other distant tissues. The bacterial components and metabolites that cross the epithelial barrier enter the circulation and may feature a systemic bioactive effect. For example, l‐carnitine found in red meat is metabolized by gut bacteria and the host liver to trimethylamine‐N‐oxide (TMAO), which promotes atherosclerosis. Consortia of bacteria of vegans and vegetarians produce less TMAO than those of omnivorous humans when given the same amount of l‐carnitine (Koeth *et al*, 2013). As in the case of TMAO, changes in the number and composition of microbes in the gut may lead to an altered signaling to the host via microbiome‐derived metabolites, mediated through (i) changes in concentration and composition of bacterially derived molecules recognized by pattern recognition receptors, (ii) changes in abundance and composition of the antigen pool available for immune system sampling, and (iii) altered concentration and composition of bacterial metabolites that influx systemically into the host (Kau *et al*, 2011; Sharon *et al*, 2014; Neis *et al*, 2015; Levy *et al*, 2017). The effect mediated by the molecules that are absorbed into the portal blood is particularly important in the liver, as it is the first organ exposed to portal blood.

In the following sections, we aim to shed light on mechanisms by which gut microbiota both positively and negatively affects development and progression of NAFLD and NASH.

## Associations between NAFLD and NASH and alterations in the gut microbiome

Several studies implicate the involvement of the gut microbiome with NASH or NAFLD in mice and humans. High‐fat diet‐fed germ‐free mice exhibit lower levels of lipids in the liver in comparison with the conventionally housed mice (Rabot *et al*, 2010). Microbiome transferred into germ free mice, from mice that developed fasting hyperglycemia and insulinemia, but not from healthy mice, led to the development of NAFLD in recipient mice (Le Roy *et al*, 2013). Using 16S rDNA profiling of mice, two bacterial species, *Lachnospiraceae bacterium 609* and *Barnesiella intestinihominis,* were found to be significantly overrepresented in the stool with a potency to induce NAFLD, while *Bacteroides vulgatus* was underrepresented in comparison with the control (Le Roy *et al*, 2013). Furthermore, a correlation analysis of NAFLD‐associated parameters and the abundance of bacterial species in mice fed with low‐fat and high‐fat diet showed association between *Lactobacillus gasseri* and *Lactobacillus taiwanensis* and the area of lipidic droplets in the liver (Zeng *et al*, 2013).

Nonalcoholic fatty liver disease and NASH in humans often co‐occur with obesity and poor dietary habits making it difficult to disentangle effects of diet and accompanying metabolic changes in liver disease from the effects mediated by the altered microbiome under these same conditions. Nevertheless, some species in humans were associated with NAFLD, with the abundance of bacterial species, such as Proteobacteria, Enterobacteria, and *Escherichia* (Zhu *et al*, 2013), or *Bacteroides* (Boursier *et al*, 2016), being higher in patients with NASH as compared to matched healthy individuals. Profiling stool microbiome of children with NAFLD showed more abundant *Gammaproteobacteria* and *Prevotella* in comparison with the microbiota of obese children without NAFLD (Michail *et al*, 2015). Of note, an increase in *Proteobacteria* and decrease in *Firmicutes* were observed during progression of NAFLD, suggesting that the gut microbiome may not be stably dysbiotic during disease progression (Loomba *et al*, 2017). Interestingly, in a dietary intervention study, NAFLD patients were subjected to low‐choline diet for 6 weeks resulting in microbiome alterations, which included *Gammaproteobacteria* correlating positively, while *Erysipelotrichia* correlating negatively with the hepatic lipid content (Spencer *et al*, 2011). The impact of nonabsorbable antibiotic treatment in modulating disease features in patients with NAFLD and NASH also supports the potential role of microbiome in its pathogenesis. Short‐term treatment of patients with steatosis and NASH with the nonabsorbable antibiotic rifaximin led to improvement in liver function (Gangarapu *et al*, 2015). Similarly, long‐term antibiotic treatment led to decrease in small intestinal bacterial outgrowth, correlating with an improvement in liver function (Madrid *et al*, 2001).

Collectively, these association studies show that correlations may exist between bacterial composition and distinct taxa and NAFLD or NASH. However, these observations are limited by the lack of reproducibility between cohorts, the absence of a mechanistic explanation for dysbiosis or of its effects on NAFLD and NASH, and lack of evidence pointing toward causality. Furthermore, in most studies microbiome is sampled from stool bacterial composition of which is distinct from the communities present in the more proximal sites of the intestine (Zmora *et al*, 2018).

## Mechanisms contributing to microbiome modulation of NAFLD and NASH

Several potential mechanisms by which gut microbiota regulates NAFLD and NASH have been studied in recent years. Suggested mechanisms include dysbiotic bacteria and their derived products translocating to the liver through a disrupted gut barrier, where they evoke a hepatic inflammatory reaction and commensal or metabolite‐induced interplays with dietary factors in inducing steatosis.

## The microbiome–gut–liver anatomical axis

**Figure 1:**
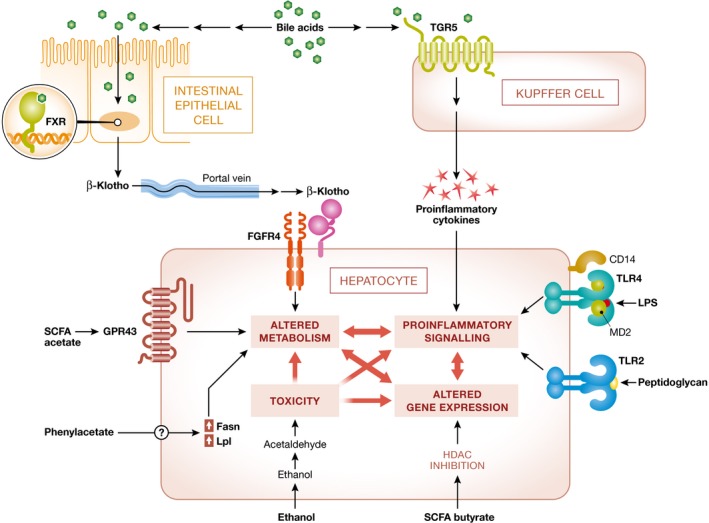
Gut microbiota‐derived compounds affecting liver metabolism Microbiome‐derived compounds affect the hepatocytes via small molecules leading to highly interconnected effects including pro‐inflammatory signaling, changes in gene expression, and alteration in metabolism and toxicity. Bile acids bind their receptor in the intestinal epithelial cells leading to release of βKlotho that travels via portal vein to the liver where it binds FGFR4 on the surface of hepatocytes and leads to changes in metabolism. Bile acids also activate the TGR5 receptor on the Kupffer cells leading to secretion of pro‐inflammatory cytokines that in turn signal to hepatocytes. Bacterial pattern molecules, i.e., peptidoglycan and LPS, signal via TRL2 and TRL4. Short‐chain fatty acids act via binding to their receptors (acetate binds GPR43) and alter metabolism; additionally, butyrate is histone deacetylase (HDAC) inhibitor and regulates gene expression through modulation of chromatin state. Phenylacetate by unknown mechanism affects expression of metabolic genes such as Fasn and Lpl leading to metabolic changes. Finally, acetaldehyde, toxic derivative of ethanol, exacerbates high oxidative stress on the hepatocytes.

The concept of gut bacteria having influence on liver homeostasis stems from the intimate anatomical interaction between the gastrointestinal tract and the liver, which is often termed “gut–liver axis”. Indeed, such connection is derived from the embryonic development, when the liver buds directly from the foregut. The liver plays a crucial role at the nexus of host–microbe interactions because it is the first organ to drain the gut through the portal vein. The portal blood, in addition to nutrients, contains other molecules that either actively or passively cross from the gut to the blood (Fig 1). This makes the liver one of the most exposed organs to intestinal bacteria and bacterial‐derived products (Macpherson *et al*, 2016). Disturbance of the gut–liver axis was shown to play a pivotal role in the pathogenesis of NAFLD. These include gut barrier disruption, bacterial translocation and inflammatory response in the liver, such as Toll‐like receptor (TLR) signaling and inflammasome activation, and changes in composition of bacterial products.

## Microbiome impact on gut barrier function

The healthy intestinal epithelium forms a tightly sealed physical barrier that separates the host from the contents of the gut. Tight junctions are one of the most important physiological and pathological regulators of intestinal permeability. Under physiological conditions, tight junction proteins, such as zonula occludens, seal adjacent epithelial cells at the apical sites and prevent bacteria from entering the intestinal mucosa and the bloodstream (Turner, 2009). However, such regulation becomes pathological with disruption of tight junctions and excessive paracellular leakage of non‐self antigens to the lamina propria, which subsequently leads to the development of NASH (Fasano, 2008). The gut barrier consists of not only tight junctional complexes but also mucus layer produced by goblet cells, antimicrobial defense mediated by Paneth cells, and the complicated network of innate and adaptive immune cell populations. A delicate crosstalk and balance among gut microbiota, intestinal epithelial cells, and gut mucosal system are important to maintain intestinal permeability and tissue homeostasis (Peterson & Artis, 2014). There is emerging evidence for a dysfunctional gut barrier or altered gut permeability in NAFLD patients (Miele *et al*, 2009; Volynets *et al*, 2012; Luther *et al*, 2015). A meta‐analysis based on five clinical studies shows that NAFLD and NASH patients are more likely to have altered gut permeability in comparison with healthy controls (Luther *et al*, 2015). The association of increased intestinal permeability is stronger particularly in NASH patients, demonstrating that the inflammatory changes observed in NASH might be caused by the increased intestinal permeability (Luther *et al*, 2015). On the molecular level, NAFLD patients exhibit decreased expression of zonula occludens‐1 (ZO‐1) and junctional adhesion molecule A (JAM‐A; Miele *et al*, 2009; Rahman *et al*, 2016).

However, the co‐occurrence of NAFLD/NASH and disruption of the gut epithelial barrier do not prove causation. Whether the alteration of gut permeability is a cause or a consequence of NAFLD is an intriguing question but remains unclear to date. The study by Luther *et al* (2015) suggested that initial liver damage might precede the development of disrupted gut permeability. Using methionine–choline‐deficient (MCD) diet‐induced NASH model *in vivo,* the authors found that the disruption of tight junction proteins and altered intestinal permeability only occurred after initial hepatic injury (Luther *et al*, 2015). However, whether this holds true in other animal models or in humans remains to be investigated.

Regardless of the triggering event, impaired gut permeability aggravates NASH. As an example, induction of a leaky and inflamed gut by dextran sulfate sodium (DSS) leads to translocation of lipopolysaccharide (LPS) to the systemic circulation and consequently worsens liver inflammation and fibrosis in mice fed with high‐fat diet (Gäbele *et al*, 2011). Similarly, JAM‐A‐deficient mice, a genetic model of gut barrier dysfunction, when fed with high‐fat, fructose, and cholesterol diet, develop more severe steatohepatitis than control mice (Rahman *et al*, 2016).

## Microbiota impact on liver steatosis

Fat absorption is impacted by the gut microbiota (Bäckhed *et al*, 2004), including the duodenal microbiota (Martinez‐Guryn *et al*, 2018), and is considered a contributing factor to liver steatosis (Le Roy *et al*, 2013). Several effector molecules have been linked to these effects.

### Short‐chain fatty acids

Complex carbohydrates, such as dietary fiber and resistant starch, are digested by many different gut bacterial species leading to release of short‐chain fatty acids (SCFAs): acetate, propionate, butyrate, pentanoic (valeric) acid, and hexanoic (caproic) acid (Høverstad & Midtvedt, 1986). Although most SCFAs are utilized in the gut, some amount is transported to the bloodstream through the monocarboxylate transporter 1 (MCT‐1) and the sodium‐coupled monocarboxylate transporter 1 (SMCT‐1) receptors and via the portal vein reach the liver, where they can be channeled into the tricarboxylic acid (TCA) cycle and become source of energy. In addition, SCFAs are also potent signaling molecules through binding to G‐protein‐coupled receptors GPR41, GPR43, and GPR109A (Samuel *et al*, 2008; Maslowski *et al*, 2009). While GPR41 binds propionate and GPR43 primarily recognizes acetate, both can also bind all other SCFAs with lower affinity (Brown *et al*, 2003). Moreover, other GPCRs, such as OLFR78 and GPR109A, can also bind SCFA with varying affinity adding complexity to the system (Pluznick *et al*, 2013; Kasubuchi *et al*, 2015). Importantly, butyrate and to some level propionate and acetate can act as histone deacetylase (HDAC) inhibitors. Acetylation of histone tails leads to introduction of negative charge and to less efficient binding of histones to the DNA, resulting in opening of the chromatin and gene expression. Hence, SCFAs acting as inhibitors of histone deacetylases positively regulate gene expression. The repertoire of genes affected depends on the cellular context. Moreover, in the liver short‐chain fatty acids regulate synthesis of cholesterol, a precursor of bile acids, which are potent mediators of communication between gut microbiome and the host (Hara *et al*, 1999).

One of the mechanisms by which short‐chain fatty acids affect fat accumulation both in the liver and in the adipose tissue is regulation of insulin sensitivity via GPR43. Short‐chain fatty acid activation of GPR43 signaling in adipose tissue promotes energy expenditure and inhibits fat accumulation in adipose tissue as well as in the liver (Kimura *et al*, 2013). Unlike colonized mice, germ‐free mice in which short‐chain fatty acids are not produced by the microbiome maintain normal weight regardless of GPR43 being knocked out (Kimura *et al*, 2013). Similarly, mice treated with antibiotics developed more severe NAFLD and had higher insulin resistance and adiposity (Mahana *et al*, 2016).

Another possible mechanism of action of short‐chain fatty acids to limit NASH is by reducing inflammatory signals. In murine models of colitis, arthritis, and asthma, deletion of GPR43 leads to increased pro‐inflammatory cytokine production and immune cell recruitment (Maslowski *et al*, 2009). Similar phenotypes are observed in germ‐free mice that lack short‐chain fatty acid‐producing bacteria (Maslowski *et al*, 2009). In humans, increases in the amount of consumed fiber lead to higher expression of GPR41 and GPR43 and lower inflammatory markers and improved lung function in patients with asthma (Halnes *et al*, 2017).

While many studies investigated the roles of SCFAs on gut physiology (Macia *et al*, 2015), their function in liver disease remains to be fully elucidated. An initial study indicated an association between NASH and low‐fiber diet (Cortez‐Pinto *et al*, 2006), while another study showed some improvement in blood alanine transaminase (ALT) and aspartate transaminase (AST) activity (measure of liver damage) and cholesterol in NAFLD patients fed on high‐fiber diet (Rocha *et al*, 2007). Interestingly, overweight and obese patients have higher level of propionate in their stool, suggesting that either it is overproduced or its absorption is disrupted (Schwiertz *et al*, 2010). Larger studies are needed to validate these observations and determine if and how SCFAs may play a role in the pathogenesis of NAFLD and NASH.

### Bile acids

Primary bile acids (BAs) are synthesized by the liver, are secreted to the gallbladder, and released into the duodenum following food ingestion. In the gut, they are metabolized to secondary bile acids by the gut bacteria. Then, they are reabsorbed into the portal vein and most of the molecules are captured by the liver and are recirculated, but some remain in the blood and act as signaling molecules. Bile acids function in the host in a dual manner: (i) they facilitate lipid solubilization, digestion, and absorption and (ii) they act as signaling molecules via, among others, TGR5 and FXR receptors (Sinal *et al*, 2000; Pols *et al*, 2011). Chemical identity and composition of the bile acid pool affect lipid absorption and potency to activate receptors.

The enzymes for dehydroxylation of the hydroxyl group at the C‐7α position of deconjugated BAs to form the secondary BAs are expressed throughout the entire bacterial kingdom, but *Clostridium XIVa* was identified to have the most potent enzymes for this conversion (Ridlon *et al*, 2013). The other BA transformation that is mediated by bacteria is the dehydrogenation and epimerization of 3‐, 7‐, and 12‐hydroxyl groups. Ridlon *et al* (2006) summarized the complex metabolism of bile acids and the reactions carried out by specific bacteria.

Patients with NAFLD feature elevated levels of bile acids in liver tissue, serum, and urine with a significantly higher ratio of more hydrophobic and cytotoxic bile acid species (Aranha *et al*, 2008; Ferslew *et al*, 2015). Perturbations in gut microbiota levels and community composition are very likely to affect the bile acid pool, but this causal relationship has not been explicitly showed yet *in vivo*. It is hypothesized that commensal microbes modulate host cellular metabolism through bile acid conversions, including lipid and glucose homeostasis, but this merits further studies. Furthermore, it is hypothesized that changes in microbiome resulting from bariatric surgeries may have beneficial effect on NASH, but the contribution of microbiome and other factors was not disentangled yet (Chavez‐Tapia *et al*, 2010).

### Choline

The effect of choline deficiency on the development of NAFLD and NASH is well established, corroborated by the fact that choline‐deficient diet is a commonly used murine model of NASH (Blumberg & McCollum, 1941). In the liver, choline is mainly used in biogenesis of phosphatidylcholine or is directed to maintain the S‐adenosyl methionine (SAM) cycle. Moreover, the liver is an important storage compartment for choline in the body. Deletion of genes involved in choline metabolism and its availability to the SAM cycle or deletion of genes of the SAM cycle leads to NAFLD. Furthermore, in the absence of choline, very‐low‐density lipoprotein (VLDL) levels are down‐regulated, due to insufficient phosphatidylcholine. The hepatic ratio of phosphatidylcholine to its unmethylated precursor phosphatidylethanolamine in NAFLD is lower than in healthy subjects, suggesting deficiency in methyl donor SAM (Arendt *et al*, 2013). Choline has two origins: exogenous and endogenous. Diet provides about 70% of choline, while the remainder is synthesized in the body. The amount of available choline depends on its amount in the diet and on its metabolism in the gut by microbiota, which is also regulated by choline levels (Spencer *et al*, 2011).

Commensal bacteria, e.g., *E. coli*,* Desulfovibrio desulfuricans*, can convert choline to methylamines such as trimethylamine (TMA), dimethylamine (DMA), and monomethylamine (MMA; Ackermann & Schutze, 1910; Zeisel *et al*, 1983; Craciun & Balskus, 2012). The choline utilization cluster, present in many different bacterial species, carries the function of metabolism of choline to TMA (Al‐Waiz *et al*, 1992; Zhang *et al*, 1999; Craciun & Balskus, 2012; Martínez‐del Campo *et al*, 2015). Germ‐free mice inoculated with bacterial isolates predicted to metabolize choline to TMA in comparison with mice inoculated with bacteria that do not metabolize choline, featured choline depletion both in blood serum and in feces (Romano *et al*, 2015). Species shown to metabolize choline to TMA *in vitro* include *Anaerococcus hydrogenalis, Clostridium asparagiforme, Clostridium hathewayi, Clostridium sporogenes, Escherichia fergusonii, Proteus penneri, Providencia rettgeri* and *Edwardsiella tarda* (Romano *et al*, 2015). Most studies focus on the role of TMA derivative TMAO, as it was shown to play role in atherosclerosis (Koeth *et al*, 2013). Additionally, dysbiotic microbiota featuring enhanced conversion of choline to methylamines can potentially lead to deficiency of choline and contribute to NASH. Indeed, it was shown that NAFLD is associated with lower levels of phosphatidylcholine and higher levels of TMA in the blood, implicating the role of microbiota in the imbalance of choline metabolic flux (Dumas *et al*, 2006).

Bacteria can also utilize choline to synthesize phosphatidylcholine via the phosphatidylcholine synthase (Pcs) pathway. In eukaryotes, phosphatidylcholine is a membrane component, but it is estimated to be present in the membranes of 10–15% of bacterial species including *L. pneumophila* and *P. aeruginosa* (Sohlenkamp *et al*, 2003; Geiger *et al*, 2013). In the context of NAFLD‐associated small intestinal bacterial bloom and overgrowth, bacterial demand for choline, aimed at synthesizing phosphatidyl choline for growth and division, may increase and contribute to choline deficiency in the host and resultant NAFLD and NASH.

### Microbial fermentation to alcohol

Microbiota harbors genes that can ferment dietary sugars into ethanol that can then enter the bloodstream. The amount of alcohol produced depends on the availability of carbohydrates from the diet and is increased in obese mice (Cope *et al*, 2000). In children with NASH as well as in adults with NAFLD, there is significantly more bacteria associated with increased alcohol levels in the blood in comparison with obese children without NASH (Volynets *et al*, 2012; Zhu *et al*, 2013; Michail *et al*, 2015).

Alcohol exacerbates oxidative stress and inflammation in the liver. Moreover, in the liver alcohol dehydrogenase metabolizes ethanol to toxic acetaldehyde, which due to its electrophilic nature forms adducts with proteins and other molecules in cells leading to loss of structure and function. Acetaldehyde is converted to acetate by CYP2E1, but this pathway can be saturated leading to accumulation of this toxic intermediate. Expression of CYP2E1 is up‐regulated in the presence of ethanol. CYP2E1 does metabolize not only acetaldehyde, but also other molecules, and its function is linked to the level of oxidative stress in the liver, which is a potent pro‐fibrotic signal. Interestingly, patients suffering from NASH exhibit increased CYP2E1 levels (Zong *et al*, 2012).

### Phenylacetate

It was recently suggested that the microbially derived metabolite of phenylalanine, phenylacetate, is up‐regulated in blood of females with NASH (Hoyles *et al*, 2018). This molecule's blood levels have higher predictive power of the disease than conventional diagnostics. Microbiota of patients with NASH is enriched with genes for phenylalanine metabolism to phenylacetate, potentially suggesting an involvement in generation of this NASH‐associated molecule. Interestingly, mice chronically treated with phenylacetate feature elevated expression of genes related to fat metabolism (*Lpl, Fasn*), increased levels of hepatic triglycerides, and eventually developed liver steatosis (Hoyles *et al*, 2018). In NAFLD and NASH patients in addition to phenylalanine, phenylacetate precursor, abundances of other amino acids, notably branched chain amino acids (BCAAs), are altered and associate with disease severity, suggesting that there may be other metabolites produced by microbiome that play role in the disease (Gaggini *et al*, 2018).

## Microbiome‐induced induction of liver inflammation

**Figure 2:**
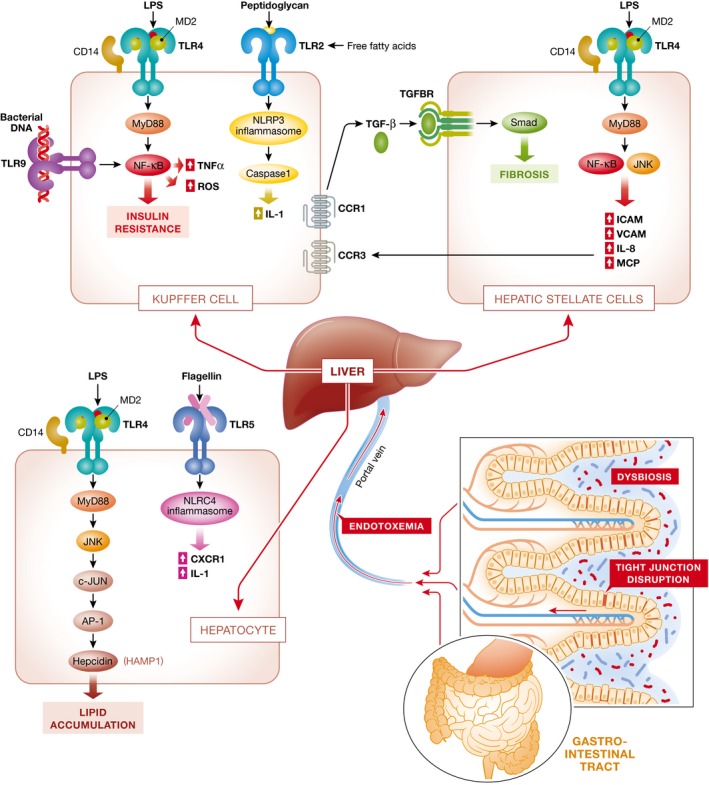
Pattern diagram featuring gut permeability, bacterial translocation, and TLR signaling in NAFLD/NASH Gut microbiota alteration might contribute to increased gut permeability, translocation of bacteria, and microbiota products from the gut to liver through the portal vein. Different TLRs (TLR4, TLR9, TLR2, TLR5) in different cell types of the liver (Kupffer cells, hepatic stellate cells, hepatocytes) sense bacterial products and trigger downstream inflammatory responses as well as cytokine production. Different cell types, such as Kupffer cells and hepatic stellate cells, might interact with each other in inducing inflammation and fibrosis.

Gut barrier disruption leads to the translocation of overgrowing bacteria and their products to mucosa and circulation, and hence initiates or enhances liver inflammation (Fig 2). There is already an increased bacterial translocation from the gut toward blood and remote tissues at the early onset of HFD‐induced type 2 diabetes, which leads to a continuous metabolic bacteremia (Amar *et al*, 2011). In this context, the most extensively studied microbial molecule is LPS, a cell wall component of gram‐negative bacteria, also known as endotoxin (Soares *et al*, 2010). Systemic LPS concentration is significantly elevated in NAFLD in both human and animal studies (Yang *et al*, 1997; Bergheim *et al*, 2008; Harte *et al*, 2010; Sharifnia *et al*, 2015). High‐fat diet in mouse models induces LPS elevation to a level, which sufficiently initiates obesity and insulin resistance, and similar metabolic responses are triggered in normal diet‐fed mice with chronic infusion of LPS (Cani *et al*, 2007). The effect of LPS on the development of NASH has also been shown in genetically obese fatty/fatty rats and obese/obese mice, which are more susceptible to LPS‐mediated liver injury (Yang *et al*, 1997).

On the molecular level, LPS, a pathogen‐associated molecular pattern (PAMP), is recognized by the specific pattern recognition receptor, called Toll‐like receptor 4 (TLR4) and its co‐receptors, LPS binding protein (LBP) and CD14. Activation of TLR4 triggers downstream inflammatory cascade, involving, depending on the context, NF‐ĸB, AP‐1, and IRF3 activation (Zhai *et al*, 2004; Abu‐Shanab & Quigley, 2010). TLR4‐deficient mice display decreased liver injury, inflammation, and lipid accumulation in comparison with wild‐type mice in NAFLD models induced by high fructose or MCD diet (Rivera *et al*, 2007; Spruss *et al*, 2009), which confirms the role of TLR4 in inducing inflammation in NAFLD. Consistently, CD14 mutant mice are resistant to LPS‐initiated metabolic features including obesity, diabetes, and liver fat accumulation and inflammation, indicating that metabolic endotoxemia triggers NAFLD in a CD14‐dependent approach (Cani *et al*, 2007).

TLR4 is expressed by the immune and parenchymal cells of the liver; hence, the effect of LPS on the liver is compounded, and disentangling it requires understanding of all the components and interactions between them. Depletion of Kupffer cells with clodronate liposomes in MCD diet‐fed mice decreases the overall TLR4 expression in the liver and blunts the histological evidence of NASH (Rivera *et al*, 2007). Kupffer cells activated by LPS produce pro‐inflammatory cytokines IL‐18, IL‐1β, and IL‐12, leading to induction of NK cells and cytotoxic T cells (Takahashi *et al*, 1996; Seki *et al*, 2001). In fructose‐induced NAFLD in mice, the activation of Kupffer cells through TLR4‐dependent mechanism is mediated via a MyD88‐dependent signaling pathway, leading to induction of hepatic TNF‐α mRNA, formation of reactive oxygen species (ROS), and insulin resistance (Rivera *et al*, 2007; Spruss *et al*, 2009). TLR4 expressed by hepatocytes responds to LPS and regulates the expression of hepcidin, a key iron‐regulatory protein implicated in obesity and NAFLD, via the MyD88‐IRAK‐TRAF6‐JNK‐AP‐1 axis (Lu *et al*, 2016; Lee *et al*, 2017). In liver sinusoidal endothelial cells (LSEC), TLR4 activation leads to induced production of TNF‐α and ROS, but *in vivo* they are mostly exhibiting tolerance to LPS and its effect is probably minimal (Sarphie *et al*, 1996; Uhrig *et al*, 2005). TLR4 signaling also plays an important role in activation of fibrogenic phenotype in hepatic stellate cells (HSCs). Activated HSCs produce various chemokines and adhesion molecules, which in turn induce chemotaxis of liver macrophages—Kupffer cells. Recruited Kupffer cells enhance TGF‐β production, which binds to TGF‐β receptor on HSCs, further activating fibrogenesis (Paik *et al*, 2003; Seki *et al*, 2007).

In addition to TLR4, other TLRs are also found to play role in the development of NASH, including TLR9, TLR5, and TLR2. The role of TLR2, a receptor for peptidoglycan, is contradictory in current studies. In some studies, TLR2 knockout enhances NASH in murine NASH models induced by methionine–choline‐deficient diet, accompanied by elevated expression of inflammatory and fibrosis markers, suggesting a protective role of TLR2‐mediated signals (Szabo *et al*, 2006; Rivera *et al*, 2010). Inversely, TLR2‐deficient mice fed with choline‐deficient amino acid (CDAA) defined diet‐suppressed progression of NASH (Miura *et al*, 2013), which is consistent with findings in high‐fat‐induced metabolic syndrome in murine models (Ehses *et al*, 2010; Himes & Smith, 2010). Indeed, TLR2 signaling and palmitic acid signaling in Kupffer cells and macrophages are both required to activate NLRP3 inflammasome and secrete IL‐1 that results in progression to NASH (Miura *et al*, 2013). The contradictory role of TLR2 might be due to different NASH models used in different studies. It is proposed that CDAA diet model is more relevant to human NASH, which is characterized by obesity, insulin resistance, and liver fibrosis (Miura *et al*, 2013). Flagellin receptor (TLR5)‐deficient mice develop metabolic syndrome including hepatic steatosis (Vijay‐Kumar *et al*, 2010). Furthermore, hepatocytes lacking TLR5 show impairment in elimination of flagellated bacteria and are more susceptible to the liver injury induced by MCD diet or high‐fat diet (Etienne‐Mesmin *et al*, 2016). In a murine model of NASH, TLR9 bacterial DNA sensing and downstream signaling up‐regulates IL‐1β production by Kupffer cells, induces chemotaxis of macrophages and neutrophils, and leads to steatosis, inflammation, and fibrosis (Miura *et al*, 2010; Mridha *et al*, 2017). In mice, administration of a TLR9 antagonist protected from NASH, but it was also suggested that it was host mitochondrial DNA rather than bacterial DNA that activated this receptor in disease (Garcia‐Martinez *et al*, 2016). The roles of other TLRs in regulating NASH remain largely unknown and needs to be further investigated.

Signals from TLRs converge at the inflammasome that orchestrates effector functions, including IL‐1β and IL‐18 secretion. So far, NLRP3 and NLRP6 were implicated to have a role in microbiome induction of NASH (Henao‐Mejia *et al*, 2012). Kupffer cells activated by palmitic acid, in a NLRP3 inflammasome‐dependent manner, secrete pro‐inflammatory IL‐1β and IL‐18, thus contributing to NASH development (Cai *et al*, 2017). Another study showed that besides Kupffer cells, inflammasome components are also present in HSCs and are required for the development of liver fibrosis (Watanabe *et al*, 2009).

## Causal evidence to microbiome involvement in the pathogenesis of NAFLD and NASH

Although only a minority of cause‐and‐effect studies linking the microbiome to NAFLD and NASH pathogenesis have been conducted to date, evidence regarding the causal role of gut microbiota in NAFLD is increasing. In contrast to conventionally raised mice, germ‐free mice fed with hypercaloric diet exhibit an impaired weight gain and lipid metabolism as well as lack of liver steatosis (Kaden‐Volynets *et al*, 2018; Martinez‐Guryn *et al*, 2018). However, conventionalization of germ‐free mice with normal mouse microbiota induces *de novo* hepatic lipogenesis, in addition to increased body fat content and insulin resistance (Bäckhed *et al*, 2004). Moreover, germ‐free mice colonized with high‐fat diet‐induced jejunal microbiota showed increased lipid absorption even on low‐fat diet (Martinez‐Guryn *et al*, 2018), suggesting the crucial role of intestinal microbiota in triggering metabolic response. Inflammasome‐deficient mice aggravate NAFLD/NASH progression via modulation of gut microbiota, and more interesting, the increased severity of NASH in these mice was transmissible through animal cohousing leading to microbiome transfer between co‐housed mice, highlighting that modulated microbiota contribute to the pathogenesis of NAFLD (Henao‐Mejia *et al*, 2012). The decisive role of microbiota in NAFLD development is further confirmed by a study where differences in microbiota composition between the NAFLD‐resistant and NAFLD‐susceptible phenotypes were identified (Le Roy *et al*, 2013). In this study, only germ‐free mice receiving NAFLD‐prone microbiota developed hyperglycemia, hyperinsulinemia, and hepatic steatosis, indicating that the tendency to develop NAFLD is transmissible through gut microbiota transplantation. Further exciting evidence comes from recent studies using human donors. Gut microbiota from obese humans induced the onset of hepatic steatosis through modulation of lipid metabolism transcriptional profiles in germ‐free mice. Furthermore, mice receiving microbiota from the same donor but after weight loss exhibit normal liver physiology (Wang *et al*, 2018). In another study, high‐fat diet‐fed germ‐free mice inoculated with microbiota of NASH patients, rather than healthy donors, showed an exacerbated NASH phenotype, as manifested by increased liver steatosis and inflammation (Chiu *et al*, 2017). Based on these promising animal models, future studies are needed to establish a definite cause–effect link between dysbiosis and human NAFLD.

## The microbiome as a potential therapeutic target in NAFLD and NASH

The targeting of gut microbiota as a noninvasive diagnostic tool that correlates with the severity of NAFLD/NASH holds promise as a future therapeutic modality in these currently cureless disorders. Initial studies suggest that the progression of NAFLD and NASH may be monitored by blood serum metabolites such as phenylacetate (Hoyles *et al*, 2018) and microbiome composition (Loomba *et al*, 2017). For more specific and sensitive diagnostic tools, multiple measurements can be integrated. Indeed, in a recent study by Hoyles *et al* (2018) data from metagenomics, liver transcriptomics, and metabolomics are integrated to shed light on the importance of microbial biomarkers as a potential diagnostic tool for metabolic and fibrotic liver diseases, which can be used in conjunction with current invasive approaches to determine stages of NAFLD. Among patients with NAFLD, the early diagnosis of hepatic fibrosis is an important but challenging clinical question. In a recent study with both discovery and validation cohorts, blood microbiota alterations are associated with liver fibrosis in obese patients, featuring increased 16S rRNA concentration and decreased bacterial diversity in the blood, pointing out that blood dysbiosis might be used as a potential noninvasive biomarker (Lelouvier *et al*, 2016). Certainly, more future studies will pave the way to microbiota diagnostics as reliable predictive markers of disease onset and progression.

A number of studies investigated the feasibility of using the intestinal microbiota as a potential therapeutic target for NAFLD, including modulation by antibiotic treatment, prebiotics, probiotics, and synbiotics. These treatment strategies have been systematically reviewed elsewhere (Ma *et al*, 2017). Such therapies are expected to attenuate the disease by altering the contribution of gut microbiota to its pathogenesis. Although many animal studies presented exciting results, reproducible human clinical trial results are in great need.

The therapeutic effect of fecal microbiota transplantation (FMT) in NASH has been suggested by a few animal studies. Zhou *et al* (2017a) reported that FMT attenuated high‐fat diet‐induced NASH in mice via beneficial regulation of gut microbiota. García‐Lezana *et al* (2018) showed that FMT restored a healthy intestinal microbiota and normalized portal hypertension in a rat model of NASH. The effect of FMT on NAFLD/NASH is just beginning to be investigated and requires more animal and human clinical studies. Notably, a few clinical trials assessing treatment of NASH patients with FMT are currently underway (NCT02469272, NTR4339).

Other potential therapeutic targets, such as anti‐LPS immunoglobulin and microbiota‐associated metabolite treatment, have been suggested by several studies but merit independent validation and mechanistic evaluation. For example, oral administration of IMM‐124E, an anti‐LPS‐enriched bovine colostrum, was suggested to alleviate chronic inflammation, liver damage, and insulin resistance associated with NASH in mice models (Adar *et al*, 2012) and a small cohort of patients with biopsy‐proven NASH (Mizrahi *et al*, 2012). Pharmaceuticals targeting bile acid dysregulation in NAFLD, including FXR agonists, PPARα agonists, ursodeoxycholic acid (UDCA), and its derivatives, have entered different phases of clinical trials, and some of them have shown promising therapeutic effects (Yu *et al*, 2018). The administration of butyrate, a SCFA, effectively ameliorates lipid accumulation and liver inflammation in animal NAFLD models, through modulation of gut microbiota and gut barrier function (Zhou *et al*, 2017b), attenuation of inducible nitric oxide synthase (iNOS) induction (Jin *et al*, 2016), and suppression of inflammatory pathways (Sun *et al*, 2018).

## Perspectives and future directions

Given the high prevalence and increasing incidence of NAFLD worldwide and its close association with gut microbiota, research focusing on microbiome provides great opportunities as well as challenges in both the pathogenesis and treatment options of NAFLD/NASH. Since most previous studies adopted 16S rRNA sequencing which provided low resolution limited to genus level, it is imperative to identify NAFLD‐related microbes at strain level using highly accurate metagenomics sequencing, which also offers functional information of intestinal microbiome. Beyond association studies, future research work will aim to elucidate the direct causative relationship between gut dysbiosis and NAFLD in both animal models and human studies, aiming at microbiota‐targeted therapeutics. Despite great advances in correlating microbiota changes with NAFLD, specific molecular mechanism underlying the host–environment–microbiome interplay involved in the development and progression of NAFLD/NASH remains largely elusive. Therefore, a deeper and further understanding of gut microbiota‐associated metabolic profile in NAFLD/NASH subjects should be discovered. This will uncover novel treatment strategies to inhibit bacterial‐derived pathways that in turn lead to disease progression.

The heterogeneous clinical features of NAFLD can be explained by incorporation of dietary factors, genetics, and interpersonal gut microbiome variability. Precision medicine as a novel clinical approach is imperative in developing therapeutic approaches in such complex diseases. In fact, recent study has shown that individual glycemic response can be accurately predicted by combining personal, dietary, and microbiome features, successfully targeting personalized nutrition (Zeevi *et al*, 2015). Therefore, a precision medicine approach based on host, diet, and microbiome profiles could facilitate risk stratification and predict the variable clinical phenotypes, diagnostic accuracy, and therapeutic response of NAFLD. The implication of gut microbiome‐based precision medicine, including personalized probiotics (Suez *et al*, 2018; Zmora *et al*, 2018) and postbiotic interventions (Thaiss *et al*, 2016), might also be an important consideration for NAFLD treatment.

Altogether, opportunities and challenges provided by microbiome research open a new window for future studies, which will hopefully elucidate the more specific role of gut microbiome in NAFLD and establish microbiota‐targeted personalized treatment approaches.

**Pending issues**

(i) Identification of alterations in microbiota composition and microbial function in NAFLD/NASH patients.
(ii) Elucidation of specific gut microbiota‐associated molecular mechanism underlying the pathogenesis of NAFLD/NASH.
(iii) Proving the directly causal role of gut microbiota alteration in NAFLD/NASH development.
(iv) Establishment of the gut microbiota‐targeted precision medicine in the treatment of NAFLD/NASH.

## Conflict of interest

Eran Elinav is a payed consultant to BiomX and DayTwo.
